# Diabetes Is a Risk Factor for Pulmonary Tuberculosis: A Case-Control Study from Mwanza, Tanzania

**DOI:** 10.1371/journal.pone.0024215

**Published:** 2011-08-30

**Authors:** Daniel Faurholt-Jepsen, Nyagosya Range, George PrayGod, Kidola Jeremiah, Maria Faurholt-Jepsen, Martine Grosos Aabye, John Changalucha, Dirk Lund Christensen, Christian Bressen Pipper, Henrik Krarup, Daniel Rinse Witte, Aase Bengaard Andersen, Henrik Friis

**Affiliations:** 1 Department of Human Nutrition, University of Copenhagen, Frederiksberg, Denmark; 2 Muhimbili Research Centre, National Institute for Medical Research, Dar Es Salaam, Tanzania; 3 Mwanza Research Centre, National Institute for Medical Research, Mwanza, Tanzania; 4 Clinical Research Centre, University of Copenhagen, Hvidovre Hospital, Hvidovre, Denmark; 5 Department of International Health, University of Copenhagen, Copenhagen, Denmark; 6 Steno Diabetes Center, Gentofte, Denmark; 7 Department of Basic Sciences and Environment, University of Copenhagen, Frederiksberg, Denmark; 8 Department of Clinical Biochemistry, Aalborg University Hospital, Aalborg, Denmark; 9 Department of Infectious Diseases, Odense University Hospital, Odense, Denmark; University of Hyderabad, India

## Abstract

**Background:**

Diabetes and TB are associated, and diabetes is increasingly common in low-income countries where tuberculosis (TB) is highly endemic. However, the role of diabetes for TB has not been assessed in populations where HIV is prevalent.

**Methods:**

A case-control study was conducted in an urban population in Tanzania among culture-confirmed pulmonary TB patients and non-TB neighbourhood controls. Participants were tested for diabetes according to WHO guidelines and serum concentrations of acute phase reactants were measured. The association between diabetes and TB, and the role of HIV as an effect modifier, were examined using logistic regression. Since blood glucose levels increase during the acute phase response, we adjusted for elevated serum acute phase reactants.

**Results:**

Among 803 cases and 350 controls the mean (SD) age was 34.8 (11.9) and 33.8 (12.0) years, and the prevalence of diabetes was 16.7% (95% CI: 14.2; 19.4) and 9.4% (6.6; 13.0), respectively. Diabetes was associated with TB (OR 2.2, 95% CI: 1.5; 3.4, p<0.001). However, the association depended on HIV status (interaction, p = 0.01) due to a stronger association among HIV uninfected (OR 4.2, 95% CI: 1.5; 11.6, p = 0.01) compared to HIV infected (OR 0.1, 95% CI: 0.01; 1.8, p = 0.13) after adjusting for age, sex, demographic factors and elevated serum acute phase reactants.

**Conclusion:**

Diabetes is a risk factor for TB in HIV uninfected, whereas the association in HIV infected patients needs further study. The increasing diabetes prevalence may be a threat to TB control.

## Introduction

The number of people living with type 2 diabetes mellitus (diabetes) is projected to double between 2000 and 2030, based on increasing life-expectancy and urbanization [1]. Furthermore, the incidence of diabetes seems to continue to increase [2], [3], due to continuing changes in life-style [4], [5].

There is evidence to suggest that diabetes increases the risk of lower respiratory tract and other infections [6]–[8]. The mechanisms are not clear, but may be through impaired cell-mediated immunity [3] as well as neutrophil function [9]–[11]. Such effects are likely to be particularly detrimental in low-income countries, where diabetes usually remains undiagnosed or untreated due to weak health systems [12], and may occur in individuals with high exposure to tuberculosis (TB) and other infectious diseases.

More than 9 million people are diagnosed with TB each year, with HIV infection and undernutrition as well-established risk factors [13]. Studies primarily from middle and high-income countries suggest that diabetes is in fact associated with increased risk of TB. However, any effect of diabetes on risk of TB and other infections is of greater concern in low-income countries. If indeed diabetes is a risk factor for primary infection with TB or progression from latent TB infection to active TB disease, then increasing diabetes prevalence in TB endemic areas will further increase the TB burden. Yet, in high TB burden countries little is known about the prevalence of diabetes, and, as recently reviewed [14], no studies on the role of diabetes for TB have been carried out in Africa. Hence, the effect of diabetes on risk of TB has not been assessed in a population with a high prevalence of HIV, a known strong competing risk factor.

With more than 70.000 new cases each year Tanzania is one of the world's 22 high TB-burden countries [15]; one half of the TB cases are co-infected with HIV [16]. As of 2009, the estimated HIV prevalence in Tanzania is 5% [17]. We conducted a case-control study in Mwanza, Tanzania, to assess the association between diabetes and pulmonary TB, while assessing the role of HIV as a potential effect modifier.

## Methods

### Ethics statement

Ethical permission was obtained from the Medical Research Coordinating committee of the National Institute for Medical Research (NIMR) in Tanzania. Study approval was given by The Danish Central Medical Ethics Committee. Written and oral information was presented to all eligible participants by the health staff before written informed consent was obtained. Written consent was obtained from parents/legal guardians of any participants under 18 years of age.

### Subjects and study design

The study was conducted from April 2006 to January 2009 in Mwanza, Tanzania. Mwanza city has approximately half a million inhabitants, and is located on the southern shores of Lake Victoria. The city is administratively divided into 21 wards, 208 sub-wards and about 500 streets. Each street is further divided into an informal communal cell with 10–20 households (ten-cell), headed by a ten-cell leader. As part of a nutrition study, patients newly diagnosed with pulmonary TB at four major health facilities in Mwanza city were invited to participate in the study. Patients below 15 years of age, pregnant or lactating women, patients terminally ill from TB or HIV (judged unlikely to survive >48 hours), patients suffering from other severe diseases, and non-residents of Mwanza City were excluded. TB patients were diagnosed and treated according to international guidelines [18].

Among 866 microscopically confirmed pulmonary TB patients enrolled in the nutritional study [19], [20], we invited 400 consecutive patients to participate as index cases in the case-control study. Controls were recruited consecutively during a limited time. During the recruitment of controls all TB patients were invited as an index case. Index cases consenting to participate in the case-control study were asked to provide detailed contact information about address and their ten-cell leader. In co-operation with the ten-cell leader a complete list of individuals from the same ten-cell, with the same sex, and similar age (+/- 5 years) as the case was made. From the list of potential controls one individual was then randomly selected using a lottery method, and invited to participate. The same inclusion and exclusion criteria used for cases were applied to controls, except that history of TB in the household of the participant as well as evidence of active TB (cough, intermittent fevers, excessive night sweating in the past two weeks, and unexplained weight loss in the past month) were exclusion criteria among controls. If the invited control was not eligible, then another was randomly selected. Both index and non-index cases were included as cases.

### Measurements

Pulmonary TB cases were initially diagnosed with sputum-positive pulmonary TB using microscopy based on the three sputum samples submission procedure (“spot-morning-spot”) collected at the local health facility. An additional early morning sputum sample was collected in a sterile universal bottle, and culture of *Mycobacterium tuberculosis* was done on Lowenstein Jensen solid media at the Zonal TB Reference Laboratory. Except for TB diagnostics, cases and controls underwent the same examinations, and the same demographic and medical history information was collected using standardised questionnaires.

Venous blood was drawn and serum collected and kept at −80°C until analysis. HIV status was determined using two rapid tests, *Determine HIV 1/2* (Inverness Medical Innovations Inc., Delaware, USA) and *Capillus HIV-1/HIV-2* (Trinity Biotech Plc., Wicklow, Ireland). If the tests were equivocal, HIV diagnosis was based on ELISA. Serum concentrations of the acute phase reactants, C-reactive protein (CRP) and alpha-1-acid glycoprotein (AGP) were determined at the Department of Clinical Biochemistry, Aalborg University Hospital, Denmark. Serum CRP was reported in mg/L and serum AGP in g/L with a lowest detection limit at 10 mg/L and 0.4 g/L, respectively. Serum CRP above 10 mg/L and AGP above 1.2 g/L were reported as elevated [21], [22]. Weight and height were measured with the participant barefooted and with minimal clothing to the nearest 0.1 kg and 0.1 cm, respectively. The body mass index (BMI) was calculated as weight/height^2^ (kg/m^2^).

Cases and controls were tested for impaired glycaemia and diabetes and diagnosed according to the most recent WHO classification [23]. The testing was performed a few days after the initiation of TB treatment, since the patients were not fasting on the day of recruitment. The diagnosis was based on fasting capillary blood glucose (FBG) and 2 hour blood glucose (2hBG) after a standard 75 g oral glucose tolerance test (OGTT). Blood glucose levels were analysed using the HemoCue Glucose System (HemoCue, Ängelholm, Sweden). Measurement of FBG and the OGTT procedure were performed in the morning. All participants were instructed to fast from midnight (>8 hours). They were only allowed to take water prior to the test. The OGTT was performed on participants with a FBG between 5.1–11.0 mmol/L. Patients with FBG values below 5.1 mmol/L were categorized as having normal glycaemia, and those with values above 11 mmol/L as having diabetes. Based on FBG and 2hBG post OGTT, the participants could further be categorized with isolated impaired fasting glucose (iIFG), isolated impaired glucose tolerance (iIGT), combined IFG/IGT or diabetes [24]. Final diabetes diagnosis was based on either a FBG >6 mmol/L or a 2hBG>11 mmol/L [23]. Participants diagnosed with diabetes were referred to the local diabetes clinic for management. In the present study, all participants were tested irrespective of prior diabetes diagnosis and only classified as having diabetes if the diagnosis could be confirmed by the study [23].

### Statistical analysis

Data were double entered, and all statistical analyses were performed using Stata 11.0 (Stata Corp., Texas, USA). Only cases and controls with complete data on glucose tolerance status were included in the analysis. Cases were only included if the sputum culture confirmed the TB diagnosis, unless culture was missing or contaminated, in which case diagnosis was based on a sputum positive microscopy. P-values <0.05 were considered significant. Blood glucose levels were analysed as continuous variables to estimate the association between blood glucose levels (FBG and 2hBG) and TB. Finally, glucose tolerance status was defined as normal glycaemia (reference group), iIFG, iIGT, combined IFG/IGT, and diabetes. Logistic regression was used to estimate the role of impaired glycaemia and diabetes as a risk factor for TB; also stratified by HIV status. In the logistic regression adjusted for age, sex, socio-demography, HIV and AGP we combined iIFG, iIGT and combined IFG/IGT (IFG/IGT). Due to a TB-related weight deficit of 9–10 kg [24], BMI and waist circumference would have functioned as nearly full proxy variables for case status in this study sample, and were consequently not included in the final analysis.

## Results

Of the 866 TB cases and 355 controls recruited, 803 (93%) and 350 (96%) had complete blood glucose data and thus included in the case-control analysis. Among TB cases 735 (92%) were culture-positive and 68 were diagnosed as sputum positive using smear microscopy due to either a contaminated or missing culture sample. There was no difference in baseline characteristics between index and non-index cases with mean age (SD) of 35.2 (11.7) and 34.5 (12.1) years (p = 0.44), respectively, and proportion of females being 40.9 and 35.2% (p = 0.10). Baseline characteristics of all cases and controls are shown in **Table 1**. Mean BMI was lower among cases, and the proportion with elevated serum acute phase reactants was higher. Anti-retroviral treatment was used by 10.7% of HIV infected.

**Table 1:** Background characteristics of 803 tuberculosis cases and 350 controls (n = 1153)^1^.

	Cases (n = 803)		Controls (n = 350)		p
Female sex (%)	38.0		45.0		0.03
Age (y)	34.8	(11.9)	33.8	(12.0)	0.19
Ethnic group (%)					
Msukuma tribe (%)	45.3		46.0		0.84
Other	54.7		54.0		
Marital status					
Single	27.1		25.4		<0.001
Married/cohabiting	53.1		68.6		
Separated/divorced/widow	19.7		6.1		
Occupation (%)					
Farmer/Fisherman	36.8		31.9		0.25
Businessman/Employed	36.4		40.5		
Other	26.8		27.6		
Religion (%)					
Christian	73.7		78.5		0.02
Muslim	22.1		20.3		
Other	4.2		1.2		
BMI (kg/m^2^)	18.4	(2.7)	22.6	(4.1)	<0.001
HIV infection (%)	43.2		10.0		<0.001
Acute phase response (elevated)					
C-reactive protein >10 mg/L (%)	96.6		3.4		<0.001
Orosomucoid >1.2 g/L (%)	97.3		2.7		<0.001

1 Data are mean (SD) or%.

Impaired glycaemia (IFG/IGT and diabetes) was found in 37.6% (95% CI: 34.2; 41.1) of cases and 21.4% (17.2; 26.1) of controls. Similarly, levels of FBG and 2hBG were higher in the case group (Table 2). The test for diabetes was done within 3 and 7 days of TB treatment initiation for 48 and 74%, respectively. There were no differences in diabetes prevalence and blood glucose levels in relation to time of testing.

**Table 2:** Distribution of impaired glycaemia and diabetes and level of blood glucose based on 803 pulmonary tuberculosis cases and 350 neighbourhood controls (n = 1153)^1^.

	Cases (%)	Controls (%)	p
	n = 803	n = 350	
Glucose intolerance status			
Normal glycaemia	62.4 (58.9;65.8)	78.6 (73.9;82.8)	<0.001
Impaired glycaemia			
Isolated impaired fasting glucose (IFG)	8.0 (6.2;10.1)	6.9 (4.4;10.0)	
Isolated impaired glucose tolerance (IGT)	6.0 (4.4;7.8)	2.6 (1.2;4.8)	
Combined IFG/IGT	7.0 (5.3;9.0)	2.6 (1.2;4.8)	
Diabetes 3	16.7 (14.2;19.4)	9.4 (6.6;13.0)	
Blood glucose levels (mmol/L)			
Fasting (n = 1153)	5.3 (1.6)	5.0 (1.0)	<0.001
2 hour post oral glucose tolerance test (n = 536) 2	7.7 (2.2)	7.1 (2.3)	<0.001

1 Data are mean (SD) or% (95% CI).

2 Oral glucose tolerance test performed if fasting blood glucose level was between 5.1–11.0 mmol/L.

3 Diabetes diagnosis was based on either a FBG >6 mmol/L or a 2hBG >11 mmol/L.

In an unadjusted logistic regression model diabetes was associated with TB (OR 2.2, 95% CI: 1.5; 3.4, p<0.001), and there was no interaction with HIV (p = 0.99). After adjustment for serum concentration of the acute phase reactant AGP and other covariates the association between diabetes and TB increased to an OR of 4.2, but only among HIV uninfected, whereas no association was found among HIV infected (interaction, p = 0.01) (**Table 3**). Adjustment with CRP instead of AGP produced similar estimates. The results were not confounded by BMI nor waist circumference (data not shown).

**Table 3:** Diabetes and all levels of IFG/IGT as predictors of pulmonary tuberculosis with odds ratio (OR) and 95% confidence interval based on 803 pulmonary tuberculosis cases and 350 neighbourhood controls (N = 1153).

	OR (95% C.I.)	OR (95% C.I.)	OR (95% C.I.)
		Model 1	Model 2
	Unadjusted	Adjusted for age, sex, socio-demography 2	Model 1 + AGP 3
HIV negative (n = 770)			
Glucose intolerance status 1			
normal glucose tolerance	ref.	ref.	ref.
IFG/IGT	2.26 (1.50;3.41)	2.34 (1.52;3.61)	2.65 (1.00;7.06)
diabetes	2.15 (1.35;3.42)	2.14 (1.32;3.46)	4.23 (1.54;11.57)
HIV positive (n = 382)			
Glucose intolerance status 1			
normal glucose tolerance	ref.	ref.	ref.
IFG/IGT	2.16 (0.73;6.38)	1.78 (0.59;5.36)	3.54 (0.60;21.05)
diabetes	1.94 (0.65;5.75)	2.05 (0.68;6.19)	0.14 (0.01;1.81)

1 Oral glucose tolerance test (OGTT) performed if fasting blood glucose level was between 5.1–11.0 mmol/L.

2 Socio-demography includes religion, marital status and occupation.

3 Interaction between diabetes and HIV, p = 0.01.

Abbreviations: IFG/IGT: all levels of impaired fasting glycaemia and impaired glucose tolerance; AGP: serum alpha-1-acid glycoprotein.

## Discussion

This study shows that diabetes is a strong risk factor for pulmonary TB. Furthermore, we found a high prevalence of impaired glycaemia and diabetes among controls, randomly selected from neighbours with the same sex and similar age as index cases, but without evidence of TB. Considering that the controls were generally poor, normal weight and young the observed prevalence of diabetes of more than 9% was surprisingly high. According to estimates by the International Diabetes Foundation (IDF), the prevalence of diabetes in Tanzanian adults between 20–79 years of age is 3.2% in 2010 [2]. Our data suggest that this in an underestimate. This increase may be due to the on-going nutritional transition, i.e. increased access to refined fat and sugar combined with reduced physical activity [25].

The observed association between diabetes and pulmonary TB is in accordance with reports from several other recent studies [26]–[28]. A review of 13 observational studies, of which only one was from a low-income country and none from Africa, found that diabetes was associated with TB regardless of study design [26]. Based on the three cohort studies included in the review, the summary estimate of the relative risk was 3.1 (95% CI: 2.3–4.3). The results of the seven case-control studies included were heterogeneous, with odds ratios ranging from 1.2 to 7.8. The only data available from sub-Saharan Africa was from a hospital-based study from Tanzania (1990) reporting a 6.5% prevalence of diabetes among hospitalized TB patients, which was then compared to a prevalence of 0.9% found in a separate community survey [29].

Both cohort and case-control studies have inherent limitations. In cohort studies, those found to have diabetes are generally under treatment, which might reduce or eliminate any association, as the effect of diabetes is likely to be mediated through hyperglycaemia. In case-control studies, a positive association may be due to reverse causality, i.e. stress-induced hyperglycaemia caused by TB. One possible explanation for the elevation in blood glucose level in TB is insulin resistance caused by severe infection [30]. In the current study we adjusted for the acute phase response to reduce its effect on blood glucose levels, and hence on the association between impaired glycaemia and TB. This approach seemed to be successful and should be used, in future case-control studies, to reduce the problem of reverse causality.

To our knowledge this is the first study investigating the association between diabetes and TB in a high HIV endemic area. HIV affects half of the TB cases and one tenth of the non-TB controls in this study population and is the strongest risk factor for progression to active TB [13]. Interestingly, we found that after adjustment for the acute phase response, HIV infection modified the association between diabetes and TB. The increase in odds ratio from two to four among the HIV uninfected could be due to the adjustment for stress-induced hyperglycaemia. HIV infected patients have impaired production of some acute phase reactants [31], and the apparent lack of association between diabetes and TB among the HIV infected after the adjustment for acute phase reactants needs to be explored in future studies. Treatment with antiretroviral treatment, which is known to cause metabolic changes [32], did not affect the results (data not shown).

The association between diabetes and TB reported in this and other case-control studies most likely reflect an elevated risk of TB among diabetes patients. Poorly controlled diabetes may impair the cell-mediated immune response and neutrophil function [9]–[11], [33], and hyperglycaemia alone may provide a better environment for bacterial growth and increased virulence of various microorganisms [6].

The strength of our study is that TB was primarily diagnosed based on culture result rather than microscopy, x-ray or clinical symptoms. Furthermore, impaired glycaemia and diabetes were based on FBG and OGTT according to the WHO epidemiological definition [23], rather than FBG alone or register data. We were therefore able to assess the relationship between impaired glycaemia and diabetes, and pulmonary TB. Furthermore, by including serum concentration of acute phase proteins in the regression models we adjusted for stress-induced hyperglycaemia. With adjustment, the magnitude of the association between IFG/IGT and TB was sustained, whereas the association between diabetes and TB increased, but only among the HIV uninfected. Using FBG and 2hBG as continuous variables in the analysis instead of categories may be more biologically correct, since cut-offs used to define diabetes are arbitrary.

### Conclusion

In view of the high prevalence of diabetes found in this study in relatively young urban Tanzanian adults, an effect of diabetes on the risk of TB is a major concern. The association is strongest among HIV uninfected, whereas the effect needs to be further investigated among HIV infected. Since diabetes is increasingly widespread, even among the poor who are more likely exposed to TB, it represents a serious threat to TB control. This underscores the need for diabetes prevention and treatment programs in low-income settings, and for integration of diabetes, HIV and TB detection and case-management [34].

## Acknowledgments

The authors would like to thank all the health staff and study participants involved in the study.
